# A Model Structure on the Category of $\text{ A}_{\infty }$-Categories with Strict Morphisms

**DOI:** 10.1007/s10485-026-09870-2

**Published:** 2026-05-04

**Authors:** Mattia Ornaghi

**Affiliations:** Dipartimento di Matematica, Università degli Studi di Milano, Via Cesare Saldini 50, 20133 Milan, Italy

**Keywords:** A-infty-categories, DG-categories, Model Categories, 14F08, 18G70, 18N40

## Abstract

We prove that the category of (strictly unital) $\text{ A}_{\infty }$-categories, linear over a commutative ring *R*, with strict $\text{ A}_{\infty }$-morphisms has a cofibrantly generated model structure. In this model structure every object is fibrant and the cofibrant objects have cofibrant morphisms. As a consequence we prove that the semi-free $\text{ A}_{\infty }$-categories (resp. resolutions) are cofibrant objects (resp. resolution) in this model structure.

## Introduction and Statement of Results

We fix a commutative ring *R*, an $\text{ A}_{\infty }$-category is a *R*-linear DG-category associative up to homotopy. In a few words, an $\text{ A}_{\infty }$-category $\mathscr {A}$ is a graded category equipped with multilinear maps

$$\begin{aligned} m^n_{\mathscr {A}}:\mathscr {A}(x_{n-1},x_n)\otimes \cdots \otimes \mathscr {A}(x_0,x_1)\rightarrow \mathscr {A}(x_0,x_n)[2-n], \end{aligned}$$

for every integer $n\ge 1$ and sequence of objects $x_0,,...,x_{n}\in \mathscr {A}$, satisfying axioms (see [13, Definition 1.1.1 (1.1)] or [16, §2.1]). Considering $m^1_{\mathscr {A}}$ as the differential, we can associate to $\mathscr {A}$ the (graded) category $H(\mathscr {A})$ whose hom spaces are given by:

1 $$\begin{aligned} H(\mathscr {A})(x,y):=\bigoplus _{n\in \mathbb {Z}}H^n(\mathscr {A}(x,y)). \end{aligned}$$

Taking two $\text{ A}_{\infty }$-categories $\mathscr {A}$ and $\mathscr {B}$, we call $\text{ A}_{\infty }$*-functor* a family of multilinear maps $\{\mathscr {F}^n \}_{n\ge 0}$ of the form

2 $$\begin{aligned} \mathscr {F}^n:\mathscr {A}(x_{n-1},x_n)\otimes \cdots \otimes \mathscr {A}(x_0,x_n)\rightarrow \mathscr {B}(\mathscr {F}^0(x_{n}),\mathscr {F}^0(x_0))[1-n] \end{aligned}$$

for every integer $n\ge 1$ and sequence of objects $x_0,...,x_{n}\in \mathscr {A}$, satisfying axioms (see [13, Definition 1.2.1 (1.2)]). There are several notions of unit in the framework of $\text{ A}_{\infty }$-categories, in this paper we consider the strictly unital $\text{ A}_{\infty }$-categories with strictly unital $\text{ A}_{\infty }$-functors (see [13, Definitions 1.1.4]). From now on, we denote by $\text{ A}_{\infty }\text{ Cat }$ the category of $\text{ A}_{\infty }$-categories with the $\text{ A}_{\infty }$-functors. Note that $\text{ A}_{\infty }\text{ Cat }$ is not complete (since it does not admit equalizers [2, Lemma 1.28]).

We say that an $\text{ A}_{\infty }$-functor $\mathscr {F}$ is a *quasi-equivalence* if it induces an equivalence of (graded) categories:

3 $$\begin{aligned} {[}\mathscr {F}]:H(\mathscr {A})\rightarrow H(\mathscr {B}) \end{aligned}$$

and an equivalence $H^0(\mathscr {F}):H^0(\mathscr {A})\rightarrow H^0(\mathscr {B})$. We denote by $\text{ Ho }(\text{ A}_{\infty }\text{ Cat})$ the homotopy category, i.e. the (Gabriel–Zisman) localization of $\text{ A}_{\infty }\text{ Cat }$ with respect to the class of quasi-equivalences.

$\text{ A}_{\infty }$-categories were introduced in the early 1990s in the context of Homological Mirror Symmetry since the Fukaya category of a symplectic manifold, comes naturally equipped with a structure of this kind. Note that the pioneers of this field, such as Kontsevich, Fukaya, Oh, Ono, Ohta, Seidel, Soibelman, etc, assumed by definition a *flatness* hypothesis on the hom-spaces of an $\text{ A}_{\infty }$-category. For example, in [6, Definition 1.1] and [7, §3.2.1] an $\text{ A}_{\infty }$-category $\mathscr {A}$ is such that the hom-spaces $\mathscr {A}(x,y)$ are graded free *R*-modules, or the base commutative ring *R* is assumed to be a field, see [11] or [17].

On the other hand, the definition of $\text{ A}_{\infty }$-category makes sense without any restriction on the hom-spaces. For this reason, more recently, many people such as Ganatra, Pardon, Shende, Oh, Tanaka started to used the term *cofibrant* $\text{ A}_{\infty }$*-category* to indicate an $\text{ A}_{\infty }$-category whose hom-spaces are h-projective DG-modules (see [8, Definition 2.6], [21, 22, Definition 1.2]). Note that, despite the name, this has nothing to do with a model structure on $\text{ A}_{\infty }\text{ Cat }$, it is well known that $\text{ A}_{\infty }\text{ Cat }$ has no model structure [2, §1.5]. The term *cofibrant* is inherited by the DG-categories. Indeed a cofibrant DG-category (in the model structure of Example 2.2), has cofibrant hom-spaces. In particular it is a h-projective DG-category.

The first goal of this paper is to give a precise definition of *cofibrant* $\text{ A}_{\infty }$*-category*, namely we provided a model structure on the category of $\text{ A}_{\infty }$-categories (taking a subset of $\text{ A}_{\infty }$-functors). In this model structure, if an $\text{ A}_{\infty }$-category is cofibrant then it has cofibrant hom-spaces, in particular it is h-projective (see Theorem A).

Despite the lack of a model structure, we can describe the hom-spaces of the homotopy category of $\text{ A}_{\infty }\text{ Cat }$ as follows:

4 $$\begin{aligned} \text{ Ho }(\text{ A}_{\infty }\text{ Cat})(\mathscr {A},\mathscr {B})\simeq \text{ Ho }(\text{ A}_{\infty }\text{ Cat})(\mathscr {A}^{\text{ hps }},\mathscr {B})/\approx . \end{aligned}$$

Here $\mathscr {A}^{\text{ hps }}$ denotes a h-projective with splits unit $\text{ A}_{\infty }$-category which is quasi-equivalent to $\mathscr {A}$ and $\approx$ denotes the weakly equivalence relation [14, Theorem A].

In order to prove that every $\text{ A}_{\infty }$-category $\mathscr {A}$ has a resolution of the form $\mathscr {A}^{\text{ hps }}$, the notion of semi-free $\text{ A}_{\infty }$-category was introduced in [14], where it was proven that the semi-free resolution of $\mathscr {A}$ is an h-projective $\text{ A}_{\infty }$-category with splits units. Note that, in the case of DG-categories, the semi-free resolutions correspond to the cofibrant resolutions in Tabuada model structure (see Example 2.2). For this reason in *loc.* *cit.* it was left as an open question if the semi-free $\text{ A}_{\infty }$-categories are a kind of *cofibrant resolutions* in an appropriate category.

We recall that an $\text{ A}_{\infty }$-functor $\textsf {F} =\{\textsf {F} ^n \}_{n\ge 0}$ is called *strict* if $\textsf {F} ^{n\ge 2}=0$, in particular $\textsf {F} ^1$ preserves the underlying graded quivers, *id est*:

$$\begin{aligned} \textsf {F} ^1(m^n_{\mathscr {A}}(a_1,...,a_n))=m^n_{\mathscr {B}}(\textsf {F} ^1(a_1),... ,\textsf {F} ^1(a_n)) \end{aligned}$$

for every $n\ge 1$ and $a_1,...,a_n\in \mathscr {A}$. We denote by $\text{ A}_{\infty }\text{ Cat}_{\textrm{strict}}$ the category of $\text{ A}_{\infty }$-categories with the strict $\text{ A}_{\infty }$-functors. In this note we prove the following:

### Theorem A

There is a cofibrantly generated model structure on $\text{ A}_{\infty }\text{ Cat}_{\text{ strict }}$ whose weak equivalences are the quasi-equivalences. In such a model structure the fibrations are the isofibrations (see Definition 3.2) strict $\text{ A}_{\infty }$-functors which are surjective on the morphisms. Every category is a fibrant object and every cofibrant object $\mathscr {A}$ is such that $\mathscr {A}(a_1,a_2)$ is a cofibrant object in $\text{ Ch }(R)$.

Despite the category $\text{ A}_{\infty }\text{ Cat }$ is not complete, $\text{ A}_{\infty }\text{ Cat}_{\textrm{strict}}$ is complete and cocomplete (see [14, Theorem 4.5]). As a consequence of Theorem A, we have that the semi-free $\text{ A}_{\infty }$-categories are cofibrant (cf. [14, Lemma D]). Moreover, consider the category *I* so defined:

### Definition 1.1

*I* is the DG-category with two objects and two closed morphisms of degree zero5such that $j_{01}\cdot j_{10}=\text{ Id}_2$ and $j_{10}\cdot j_{01}=\text{ Id}_1$.

It is not difficult to prove that, given an $\text{ A}_{\infty }$-category $\mathscr {A}$, the *path object* of $\mathscr {A}$

$$\begin{aligned} P(\mathscr {A}):=\text{ A}_{\infty }\text{ Cat }(I,\mathscr {A}) \end{aligned}$$

described in [14, 2.5] is exactly the same path object of $\mathscr {A}$ in $\text{ A}_{\infty }\text{ Cat}_{\text{ strict }}$, with the model structure of Theorem A. Cf. also [20, §3] and [20, Remark 2.7].

We conclude by saying that, given an $\text{ A}_{\infty }$-category $\mathscr {A}$, we can take the cofibrant resolution $\mathscr {A}^{\text{ cof }}$ (with respect to the model structure of Theorem A) which is cofibrant in the sense of Ganatra, Pardon, Shende et al.

### State of Art

By the work of Lefevré-Hasegawa [12] the category $\text{ Alg}_{\infty }$ of (non unital) $\text{ A}_{\infty }$-algebras linear over a field, has a model structure whose weak equivalences are the quasi-isomorphisms. In this model structure a morphism *f* is a fibration (resp. cofibration) if $f^1$ is surjective (resp. injective).

Note that $\text{ Alg}_{\infty }$ has no equalizers (see [2]) and coproducts. To see that $\text{ Alg}_{\infty }$ has no coproduct we use the fact that (the bar-cobar functor) $\text{ U }:\text{ Alg}_{\infty }\rightarrow \text{ DG-Alg }$ is right adjoint to the inclusion (see [3]). Since the tensor product is the coproduct of two DG-algebras, we must have $\text{ U }(A)\otimes \text{ U }(B)\simeq \text{ U }(A\otimes B)$, for any $\text{ A}_{\infty }$-algebras. Using this fact, it is easy to see that $\text{ Alg}_{\infty }$ has no coproduct (it has to do with the fact that there is no a good notion of tensor product of $\text{ A}_{\infty }$-algebras [15]). This is no longer true if we consider the category of $\text{ A}_{\infty }$-categories, namely, it has coproducts (which is the disjoint union) but it does not have equalizers [2, Lemma 1.28].

On the other hand, if we consider the category of $\text{ A}_{\infty }$-algebras with strict $\text{ A}_{\infty }$-morphisms we have a model structure by [9, 2.2.1. Theorem]. In this model structure the fibrations are the morphisms *f* such that $f^1$ is surjective.

Moreover, in [4] we proved that the category of $\text{ A}_{\infty }$-categories, linear over a field, is a fibrant category. The fibrations are the $\text{ A}_{\infty }$-functors $\mathscr {F}$ which are isofibrations and such that $\mathscr {F}^1$ is degree-wise surjective.

We conclude by saying that we do not known if $\text{ Alg}_{\infty }$ and $\text{ A}_{\infty }\text{ Cat }$ have coequalizers. This is an interesting question since, if it was true (at least in some cases i.e. along the cofibrations), one could try to prove that the category $\text{ A}_{\infty }\text{ Cat }$ (linear over a commutative ring) has a structure of cofibrant category.

## Model Structures and Recognition Theorem

We give two examples of model structures cofibrantly generated. It will be crucial the following result which goes under the name of *Recognition Theorem* and corresponds to [10, Theorem 2.1.19]:

### Theorem 2.1

(Recognition Theorem) Let $\mathscr {C}$ be a complete and cocomplete category with $\mathscr {W}$ a subcategory of $\mathscr {C}$, and *I* and *J* sets of maps of $\mathscr {C}$. There is a cofibrantly generated model structure on $\mathscr {C}$ with *I* as the set of generating cofibrations, *J* as the set of generating trivial cofibrations, and $\mathscr {W}$ as the subcategory of weak equivalences, if and only if the following conditions are satisfied:

1. The subcategory $\mathscr {W}$ has the two out of three property and is closed under retracts.
2. The domains of *I* are small relative to *I*-cell.
3. The domains of *J* are small relative to *J*-cell.
4. *J*-cell $$\subset $$ $$\mathscr {W}\cap I\text{-cof }$$.
5. *I*-inj $$\subset $$ $$\mathscr {W}\cap J\text{-inj }$$.
6. Either $$\mathscr {W}\cap I\text{-cof }\subset J\text{-cof }$$ or $$\mathscr {W}\cap J\text{-inj }\subset I\text{-inj }$$.

We recall that $f\in I\text{-inj }$ if *f* has the right lifting property with respect to any morphism in *I*. We say that $f\in I\text{-cof }$ if *f* has the left lifting property for any *I*-injective morphism.

### Example 2.1

We denote by $\text{ Ch }(R)$ the category of unbounded chain complexes over a commutative ring *R*. The category $\text{ Ch }(R)$ has a model structure whose weak-equivalences are the quasi-isomorphisms (see [10, Definition 2.3.3 and Theorem 2.3.11]). In order to define such a model structure we define the chain complexes $\mathbb {S}^{n-1}$ and $\mathbb {D}^{n}$. Fixed an integer *n*,where *R* is in degree $-n+1$, andwhere the only nonzero components are in degree $-n$ and $-n+1$.

The set of generating cofibrations *I* is given by the maps $i_n:\mathbb {S}^{n-1}\rightarrow \mathbb {D}^n$, for each $n\in \mathbb {Z}$. The set of generation trivial cofibrations *J* consists of $j_n:0\rightarrow \mathbb {D}^n$, for each $n\in \mathbb {Z}$. In this model structure the fibrations are the maps *f* such that $f_n$ is surjective for all $n\in \mathbb {Z}$ and the cofibrant objects are the h-projective degreewise projective modules (see [1, Theorem 9.6.1 ($\text{ ii}^{\prime }$) iff ($\text{ v}^{\prime }$)]).

Before continuing, we define by $\mathcal {K}$ the Kontsevich category:

### Definition 2.1

$\mathcal {K}$ is the DG-category with two objects generated by the following morphisms:with the relations:

1. $$d(r_{12})=r_2\cdot f - f\cdot r_1$$.
2. $$d(r_1)=g\cdot f-\text{ Id}_1$$.
3. $$d(r_2)=f\cdot g-\text{ Id}_2$$.

Note that $\mathcal {K}$ is a semi-free resolution of the DG-category *I*, where *I* is defined in (1.1); see [5, 3.7.6. Remark]. To prove that $\mathcal {K}$ is semi-free, we consider the filtration:Here

1. $I_0$ is the discrete category with two objects: 1 and 2.
2. $I_1$ is the DG-category freely generated by two closed generators *f* and *g*
3. $I_2$ is the DG-category freely generated on $I_1$ by the generators $r_1$ and $r_2$, of degree 1, such that $d(r_1)=g*f -\text{ Id}_1$ and $d(r_2)=f*g -\text{ Id}_2$.
4. $I_3$ is the DG-category freely generated on $I_2$ adding the generator $r_{12}$ and taking the quotient by the DG-ideal $(d(r_{12})-r_2*f + f *r_1)$.

We have the DG-functors $\Psi _0:I_0\rightarrow I$, $\Psi _1:I_1\rightarrow I$ and $\Psi _2:I_2\twoheadrightarrow I$, $\Psi :K\twoheadrightarrow I$. In particular, $\Psi _2$ and $\Psi$ are surjective on the morphisms and $\Psi$ is a quasi-equivalence. In particular $\Psi (j_{01})=f$, $\Psi (j_{10})=g$ and $\Psi (r_1)=\Psi (r_2)=\Psi (r_{12})=0$.

### Remark 2.1

Note that we added $r_{12}$ in $I_3$, since

6 $$\begin{aligned} r_2*f - f*r_1 \end{aligned}$$

is a closed morphism which vanish in *I* via $\Psi$. It means that, if we want to make $\Psi$ a quasi-equivalence then (6) must be a coboundary in cohomology.

On the other hand, also

7 $$\begin{aligned} r_1*g - g *r_2 \end{aligned}$$

is a closed morphism whose image via $\Psi _2$ is 0. Nevertheless we do not need to add a new generator in $I_3$, since (7) is the differential of the following morphism:

$$\begin{aligned} (g*r_{12}*g + r_1*g*r_2 - g*r_2*r_2 + r_1*r_1*g). \end{aligned}$$

### Example 2.2

In [18], Tabuada proved that the category of DG-categories has a cofibrantly generated model structure whose weak-equivalences are the quasi-equivalences. The set of generating cofibrations *I* consists of the DG-functors $\textsf {Q}$ and $\textsf {S} (n)$ described as follows. First, we denote by $\mathcal {A}$ the DG-category:$\textsf {Q}$ is the (only) DG-functor

8 $$\begin{aligned} \textsf {Q} :\emptyset \rightarrow \mathcal {A} \end{aligned}$$

from the empty set (which is the initial category of DG-cats and $\text{ A}_{\infty }\text{ Cat }$). Fixed an integer *n*, $\mathcal {C}(n)$ and $\mathcal {P}(n)$ are the two DG-categories having two objects $C_1$, $C_2$ and $P_1$, $P_2$ and whose hom-spaces are given by

9 $$\begin{aligned} \mathcal {C}(n)(C_1,C_2):=\mathbb {S}^{n-1} \end{aligned}$$

and

10 $$\begin{aligned} \mathcal {P}(n)(P_1,P_2):=\mathbb {D}^{n}, \end{aligned}$$

$\textsf {S} (n)$ is the DG-functor

$$\begin{aligned} \textsf {S} (n):\mathcal {C}(n)&\rightarrow \mathcal {P}(n)\\ \mathbb {S}^{n-1}&\mapsto \mathbb {D}^{n}. \end{aligned}$$

Now we define the trivial cofibrations. First, we denote by $\mathcal {B}$ the DG-category which has two objects $B_1$ and $B_2$ and no non trivial morphisms.

Fixed an integer *n*, the DG-functor $\textsf {R} (n):\mathcal {B}\rightarrow \mathcal {P}(n)$ is defined as follows:11The functor $\textsf {F} :\mathcal {A}\rightarrow \mathcal {K}$ is the DG-functor sending the object *A* of $\mathcal {A}$ to the object 1 of $\mathcal {K}$.

The trivial cofibrations are generated by $J:=\{\textsf {F} , \textsf {R} (n)\}$. In this model structure the fibrations are the isofibrations (see Definition 3.2) which are degreewise surjective [19, Proposition 1.13]. Moreover, every object is fibrant and the cofibrant objects are such that the hom-spaces are cofibrant in Ch(*R*), see [23, Proposition 2.3 (3)].

## Proof of Theorem A

In this section we prove the main result using the Recognition Theorem 2.1.

First, we want to find an appropriate “$\mathcal {K}$” (defined in Definition 2.1) in the case of $\text{ A}_{\infty }$-categories. We recall that, given an $\text{ A}_{\infty }$-category $\mathscr {A}$, we can take a semi-free resolution of $\mathscr {A}$. In [14, §5], we give a procedure to find such a resolution. We define

### Definition 3.1

The category $\mathcal {K}^{\text{ A}_{\infty }}$ is the $\text{ A}_{\infty }$ semi-free resolution of the category *I* (see Definition 1.1) defined as follows:

0. $I_0$ is the discrete (strictly unital) category with two objects: 1 and 2.
1. $I_1$ is the $\text{ A}_{\infty }$-category freely generated by the closed morphisms $j_{12}$ and $j_{21}$.
2. $I_2$ is the $\text{ A}_{\infty }$-category freely generated on $I_1$ adding the generators $r_1$ and $r_2$, such that
  (i).
  (ii).

Note that:As we already noted, this is not the same in the case of DG-categories, since

$$\begin{aligned} d_{\mathcal {K}}( r_1*f - f *r_2 )&= \big ( d(r_1)*f) - (f *d(r_2)) \big )\\&= (f*g*f) - (f*g *f )\\&=0. \end{aligned}$$

This is why we need to add the free generator $r_{12}$ in $\mathcal {K}$ which is the coboundary of the cocycle $r_1*f - f *r_2$. Nevertheless $\mathcal {K}^{\text{ A}_{\infty }}$ and $\mathcal {K}$ are weakly equivalent since both are h-projective with splits unit and quasi-equivalent to *I* (see [14, Theorem 5.2] and [3, Definition 3.4]). It is important to say that, since $\mathcal {K}^{\text{ A}_{\infty }}$ is h-projective, $I(n,m)=(R,0)$ is a free *R*-module (for any $n,m=\{ 1,2 \}$) and they are quasi-equivalent, then $(\mathcal {K}^{\text{ A}_{\infty }},m^1_{\mathcal {K}^{\text{ A}_{\infty }}})$ is homotopy equivalent to the complex (*R*, 0). In particular, since $\mathcal {K}^{\text{ A}_{\infty }}$ has split unit, it implies that the short exact sequence of graded complexessplits.

### Theorem 3.1

Let $\textsf {F} '$ to be the strict $\text{ A}_{\infty }$-functor from $\mathcal {A}$ to $\mathcal {K}^{\text{ A}_{\infty }}$ such that $\textsf {F} '(A):=1$. The category $\text{ A}_{\infty }\text{ Cat}_{\text{ strict }}$ has a model structure cofibrantly generated by $J':=\{ \textsf {F} ',\textsf {R} (n)\}$ (defined in (11)) and *I* (defined in Example 2.2).

First we give two definitions and a Lemma.

### Definition 3.2

An $\text{ A}_{\infty }$-functor (resp. a DG-functor) $F:\mathscr {A}\rightarrow \mathscr {B}$ is an *isofibration* if, given $a\in \mathscr {A}$ and an isomorphism $g:\mathscr {F}(a)\rightarrow b$ in $H^0(\mathscr {B})$, there exists an isomorphism $f:a\rightarrow a'$ in $H^0(\mathscr {A})$, such that $\mathscr {F}^1(f)=g$.

### Definition 3.3

An $\text{ A}_{\infty }$-functor (resp. a DG-functor) $\mathscr {F}:\mathscr {A}\rightarrow \mathscr {B}$ is *surjective* if, $\mathscr {F}^0$ is surjective (as a map of sets) and $\mathscr {F}^1$ is a surjective quasi-isomorphism of complexes.

We denote by Surj the set of strict surjective $\text{ A}_{\infty }$-functors. The following Lemma which will be useful also to characterize the fibrations of the model structure provided by Theorem 3.1.

### Lemma 3.2

A strict $\text{ A}_{\infty }$-functor $\mathscr {F}:\mathscr {C}\rightarrow \mathscr {D}$ has right lifting property with respect to

(F1): $\textsf {R} (n)$ if and only if $\mathscr {F}^1$ is surjective on the morphisms.
($\text{ F1}^{\prime }$): $\textsf {S} (n)$ if and only if $\mathscr {F}^1$ is a quasi-isomorphism surjective on the morphisms.
(F2): $\textsf {F}$ if and only if $\mathscr {F}^1$ is an isofibration.
(F3): $\textsf {Q}$ if and only if $\mathscr {F}^0$ is surjective (as a map of sets).

### Proof

Suppose that $\mathscr {F}$ is surjective on the morphisms, consider the diagram below:It is easy to find a $\tilde{\textsf {T} }$ since $\textsf {P} (n)$ is surjective on the morphisms and $\textsf {P} (n)$ is uniquely determined by the image of $\textsf {T} (R[1]\oplus R)$.

Suppose now that $\mathscr {F}$ has the right lifting property with respect to $\textsf {R} (n)$. For every morphism $g\in \mathscr {D}$ we take the functor

$$\begin{aligned} \textsf {T} :R[1]\oplus R&\rightarrow \mathscr {D}\\ (a,b)&\mapsto a\cdot g + b\cdot dg . \end{aligned}$$

If there exists $\tilde{\textsf {T} }:\mathcal {P}(n)\rightarrow \mathscr {C}$ such that

$$\begin{aligned} \mathscr {F}\big (\tilde{\textsf {T} }((a,b))\big )=\textsf {T} \big ( (a,b)\big )=a\cdot g + b\cdot dg . \end{aligned}$$

Taking $\tilde{\textsf {T} }(1,0)\in \mathscr {C}$, we have $\mathscr {F}(\tilde{\textsf {T} }(1,0))=\textsf {T} ((1,0))=g$, so $\mathscr {F}$ is surjective on morphisms.

Now we prove $\text {F1}^{\prime }$). We note that a diagram of the form:12corresponds to the datum

13 $$\begin{aligned} \{(f,\tilde{g})\in \mathscr {C}\times \mathscr {D} \text{ such } \text{ that: } \text{ deg }(f)=-n+1, m^1_{\mathscr {C}}(f)=0 \text{ and } \mathscr {F}^1(f)=m^1_{\mathscr {D}}(\tilde{g})\}. \end{aligned}$$

Moreover, the existence of a lift $\tilde{\textsf {T} }$, corresponds to the datum

14 $$\begin{aligned} \{\tilde{f}\in \mathscr {C} \text{ such } \text{ that }\, m^1_{\mathscr {C}}(\tilde{f})=f\, \text{ and }\, \mathscr {F}^1(\tilde{f})=\tilde{g}\}. \end{aligned}$$

Suppose that every diagram of the form (12) has a lift, we show that $\mathscr {F}^1$ is a surjective quasi-isomorphism. First, we prove that $[\mathscr {F}^1]$ is surjective. Let $\tilde{g}\in \mathscr {D}$ be a closed morphism, the pair $(0,\tilde{g})$ gives rise to a diagram of the form (12). Since it admits a lift then, by the characterization (14), there is a closed morphism $\tilde{f}$ such that $\mathscr {F}^1(\tilde{f})=\tilde{g}$.

Now we prove that $\mathscr {F}^1$ is surjective. Given a morphism $g\in \mathscr {D}$, we can take the datum $(0,m^1_{\mathscr {D}}(g))$, we just shown that there exists a closed morphism $\tilde{f}\in \mathscr {C}$ such that $\mathscr {F}^1(\tilde{f})=m^1_{\mathscr {D}}(g)$. Using the characterization (13), the pair $(\tilde{f},g)$ gives rise to a new diagram of the form (12). Since it has a lift, then it exists $f\in \mathscr {C}$ such that $m^1_{\mathscr {C}}(f)=\tilde{f}$ and $\mathscr {F}^1(f)=g$, and we are done.

We prove that $[\mathscr {F}^1]$ is injective. Suppose that *f* is a closed morphism in $\mathscr {C}$ such that $\mathscr {F}^1(f)$ vanishes in cohomology. It means that there exists *g* such that $\mathscr {F}^1(f)=m^1_{\mathscr {D}}(g)$. Then (*f*, *g*) forms a diagram of the form (12). Since it is liftable, there exists $\tilde{f}$ such that $m^1_{\mathscr {C}}(\tilde{f})=f$ so *f* is zero in cohomology.

To conclude, we need to prove that, if $\mathscr {F}^1$ is a surjective quasi-isomorphism, then every diagram of the form15has a lift. Using the characterization (13), we have $(f,\tilde{g})$ such that $m^1_{\mathscr {C}}(f)=0 \text{ and } \mathscr {F}^1(f)=m^1_{\mathscr {D}}(\tilde{g})$. We consider the short exact sequence of chain complexes:16Since $\mathscr {F}^1$ is a quasi-isomorphism then the cohomology of $\text{ Ker }(\mathscr {F}^1)$ is trivial. Moreover $\mathscr {F}^1$ is surjective, so we can take a morphism $h\in \mathscr {C}(x,y)$ such that $\mathscr {F}^1(h)=\tilde{g}$. We note that the morphism $m^1_{\mathscr {C}}(h)-f\in \mathscr {C}$ is closed and $\mathscr {F}^1(m^1_{\mathscr {C}}(h)-f)=m^1_{\mathscr {D}}(\tilde{g})-\mathscr {F}^1(f)=0$, so $m^1_{\mathscr {C}}(h)-f\in \text{ Ker }(\mathscr {F}^1)$. To conclude, since the cohomology of $\text{ Ker }(\mathscr {F}^1)$ is trivial, it exists $j\in \text{ Ker }(\mathscr {F}^1)$ such that $m^1_{\mathscr {C}}(j)=m^1_{\mathscr {C}}(h)-f$. Thanks to the characterization (14), the morphism $\tilde{f}=h-j$ corresponds to a lift of (15) and we are done.

To prove F2), note that every strict $\text{ A}_{\infty }$-functor $\mathcal {K}^{\text{ A}_{\infty }}\rightarrow \mathscr {D}$ is (uniquely) determined by the image of the generators. Suppose that $\textsf {F}$ has right lifting property with respect to $\mathscr {F}$, so for every commutative diagram of the form:there exists a lift $\tilde{\textsf {T} }$.

Suppose there exists $g:\mathscr {F}^0(c)\rightarrow d \in H^0(\mathscr {D})$. It means that there exist $g^{-1}$, *h*, *t* such that:

$$\begin{aligned} m_{\mathscr {D}}^2(g,g^{-1})=\text{ Id }+m_{\mathscr {D}}^1(h) \end{aligned}$$

and

$$\begin{aligned} m_{\mathscr {D}}^2(g^{-1},g)=\text{ Id }+m_{\mathscr {D}}^1(t). \end{aligned}$$

Then we can take the functor $\textsf {T} :K^{\text{ A}_{\infty }}\rightarrow \mathscr {D}$ so defined:

$$\begin{aligned} \textsf {T} (j_{12})=g \text{, } \textsf {T} (j_{21})=g^{-1} \text{, } \textsf {T} (r_1)=t \text{ and } \textsf {T} (r_2)=h. \end{aligned}$$

Since $\textsf {F}$ has a right lifting property, there exists $\tilde{\textsf {T} }:\mathcal {K}^{\text{ A}_{\infty }}\rightarrow \mathscr {C}$. It implies that there exists an isomorphism $\tilde{\textsf {T} }(j_{12})=:f$ and an object $\tilde{\textsf {T} }(2)=:c'$ in $H^0(\mathscr {C})$ such that $\mathscr {F}^1(f)=\textsf {T} (j_{12})=g$, and we are done. The proof of viceversa is similar.

Now we prove F3). We consider the commutative diagram:since every functor from $\mathcal {A}$ is uniquely determined by the image of the object *A*. It is easy to see that if there exists a lifting $\tilde{\textsf {T} }$ for every *T* then $\mathscr {F}$ must be surjective on the objects. On the other hand, if $\mathscr {F}^0$ is surjective then we can find a $\tilde{\textsf {T} }$ for every *T*. $\square$

### Proof of Theorem 3.1

We want to use Theorem 2.1, so we must verify the six conditions of the theorem.

1. 2. 3. Straightforward.
4. First we note that *I*-inj $=$ $\mathscr {W}\cap$ *J*-inj $\subset$ *J*-inj. It implies that *J*-cof $\subset$ *I*-cof and *J*-cell $\subset$ *J*-cof $\subset$ *I*-cof. So it remains to prove that, for every $\text{ A}_{\infty }$-category $\mathscr {M}$ and $\star \in J'$, the strict $\text{ A}_{\infty }$-functor $\text{ inc }$, fitting the pushout diagram 17 is a quasi-equivalence. We consider the push-out diagram: 18

We denote $N(A)\in \mathscr {M}$ by *z*. The push-out $\mathscr {P}$ is obtained by taking the disjoint union of $\mathscr {M}$ and $\mathcal {K}^{\text{ A}_{\infty }}$ and gluing the object *z* of $\mathscr {M}$ with the object 1 of $\mathcal {K}^{\text{ A}_{\infty }}$. See the following picture:

We have:

19 $$\begin{aligned} \mathscr {P}(x,y):=\bigoplus _{m\ge 0}\mathscr {P}^{(m)}(x,y), \end{aligned}$$

where

$$\begin{aligned} \mathscr {P}^{(m)}(x,y)= \underbrace{\mathscr {M}(z,y)\otimes \overline{\mathcal {K}}^{\text{ A}_{\infty }}(z,z) \otimes \mathscr {M}(z,z)\otimes ...\otimes \overline{\mathcal {K}}^{\text{ A}_{\infty }}(z,z)\otimes \mathscr {M}(x,z)}_{m\, {\text {factors}}\, \overline{\mathcal {K}}^{\text{ A}_{\infty }}}. \end{aligned}$$

Here $\overline{\mathcal {K}}^{\text{ A}_{\infty }}(z,z)$ is the chain complex:

20 $$\begin{aligned} \overline{\mathcal {K}}^{\text{ A}_{\infty }}(z,z):={\mathcal {K}}^{\text{ A}_{\infty }}(1,1)/R\cdot 1_1. \end{aligned}$$

Note that we take the quotient complex (20) because we “glue” the identity of the object $z\in \mathscr {M}$ with the identity of the object $1\in \mathcal {K}^{\text{ A}_{\infty }}$. Note that the chain complex $(\mathcal {K}^{\text{ A}_{\infty }}(1,1),m^1_{\mathcal {K}^{\text{ A}_{\infty }}})$ is homotopy equivalent to (*R*, 0) and $\overline{\mathcal {K}}^{\text{ A}_{\infty }}(z,z)$ is contractible. So

$$\begin{aligned} \text{ inc }:\mathscr {M}(x,y) \rightarrow \mathscr {P}(x,y) \end{aligned}$$

is a quasi-isomorphism. It is also clear that $H^0(\text{ inc})$ is essentially surjective since $N'(2)$ is quasi-isomorphic to $N'(1)=N(A)=z$.

**Fig. 1:**
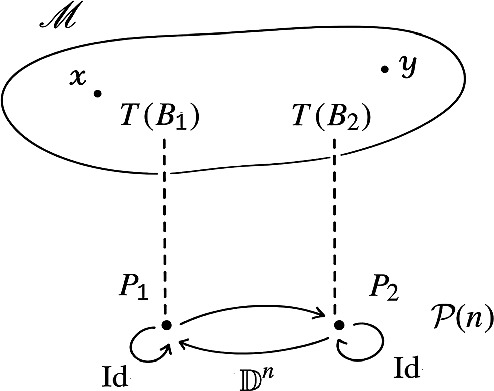
The push-out $\mathscr {P}$

On the other hand, we consider the push-out diagram:21The category $\mathscr {P}$ is given by the disjoint union of $\mathscr {M}$ and $\mathcal {P}(n)$, and by gluing the object $T(B_1)$ with the object $P_1$, and the object $T(B_2)$ with the object $P_2$. See Fig. 1.

We have:

$$\begin{aligned} \mathscr {P}(x,y)=\bigoplus _{m\ge 0}\mathscr {P}^{(m)}(x,y), \end{aligned}$$

where

$$\begin{aligned} \mathscr {P}^{(m)}(x,y)=\underbrace{\mathscr {M}(T(B_2),y)\otimes \mathbb {D}^n\otimes \mathscr {M}(T(B_2),T(B_1))\otimes ...\otimes \mathbb {D}^n\otimes \mathscr {M}(x,T(B_1))}_{m\, {\text {factors}}\, \mathbb {D}^n}, \end{aligned}$$

Since $\mathbb {D}^n$ is contractile then inc is a quasi-equivalence and we are done.

5. 6. We claim that Surj $=$ *I*-inj $=$ *J*-inj $\cap \mathscr {W}$. To prove of Surj $=$ *I*-inj we can use Lemma 3.2 item $\text {F1}^{\prime }$) and F3). Note that it is the same of [19, Lemme 1.11], since the set of generating cofibrations *I* is the same of Example 2.2. Now we want to prove *J*-inj $\cap \mathscr {W}$ $=$ Surj. If $f\in J\text{-inj }\cap \mathscr {W}$ then *f* has right lifting property with respect to $\textsf {R} (n)$ so, by Lemma 3.2 item F1), it is surjective on the morphisms. For the item F3) of the same Lemma it is an isofibration. Since it is a quasi-equivalence it is surjective on the objects. On the other hand if $f\in \text{ Surj }$, then $\textsf {R} (n)$ has the right lifting property with respect to *f* (see item F1) of Lemma 3.2). Moreover $\textsf {F}$ has the right lifting property with respect to *f* since $\mathcal {K}^{\text{ A}_{\infty }}$ is semi-free (see [14, Lemma 6.6]) and we are done.

$$\square $$

### Corollary 3.3

The fibrations are the isofibrations $\textsf {F}$ such that $\textsf {F} ^1$ are degreewise surjective. Every $\text{ A}_{\infty }$-category is fibrant.

### Proof

It follows directly from Lemma 3.2, every $\text{ A}_{\infty }$-category is fibrant since the terminal object of $\text{ A}_{\infty }\text{ Cat}_{\textrm{strict}}$ is the category with one object and one (trivial) morphism $\mathscr {A}$ (defined Example 2.2). $\square$

### Theorem 3.4

If $\mathscr {A}$ is a cofibrant $\text{ A}_{\infty }$-category then $\mathscr {A}(x,y)$ is a cofibrant object in Ch(*R*).

### Proof

The proof is the same as in the case of DG-categories (see [23, Proposition 2.3 (3)]) since they have the same set of generating cofibrations *I* of Example 2.2. $\square$

As in the case of DG-categories not all the h-projective $\text{ A}_{\infty }$-categories are cofibrant object in this model structure. For example the category $\mathcal {C}(1)\otimes \mathcal {C}(1)$ is not a cofibrant object. It is not hard to prove that the map $\emptyset \rightarrow \mathcal {C}(1)\otimes \mathcal {C}(1)$ does not have the right lifting property with respect to the trivial fibrations (see [24, Exercise 14 4.]).

## Data Availability

No datasets were generated or analysed during the current study.
